# Research Progress in High-Performance Magnesium Alloy and Its Applications

**DOI:** 10.3390/ma16155460

**Published:** 2023-08-04

**Authors:** Di Wu, Jinguo Li

**Affiliations:** 1 Shi-Changxu Innovation Center for Advanced Materials, Institute of Metal Research, Chinese Academy of Sciences, 72 Wenhua Road, Shenyang 110016, China; dwu@imr.ac.cn; 2 School of Materials Science and Engineering, University of Science and Technology of China, 72 Wenhua Road, Shenyang 110016, China

Magnesium is abundant in the Earth’s crust and seawater. Mg alloy is the lightest metallic structural material, with the advantages of high specific strength, high specific stiffness, good electromagnetism shield, good damping capacity, good machinability, easy recycling, etc. Therefore, it has extremely broad application prospects and has drawn considerable interest in the automobile, electronics, electrical appliance, transportation, aerospace, aviation, and the national defense military industries. This Special Issue (SI), “Research Progress in High-Performance Magnesium Alloy and Its Applications”, presents recent developments and excellent results in the field of Mg alloys, and includes ten articles covering some interesting and hot aspects of the topic. The purpose of the current Editorial is to briefly summarize the publications included in this SI.

Plastic processing is a promising method for improving the mechanical properties of metallic materials. Wrought Mg alloy usually exhibits superior strength and shows ample application potential. However, Mg alloy seems inferior in plastic deformation due to its HCP crystal structure, making it unable to provide sufficient independent slip systems. This SI fortunately covers the recent advances in the regulation of microstructure and mechanical properties during three traditional plastic processes, i.e., rolling, extruding, and forging. Twinning is the crystallographic shear process in grain which can change the grain orientation for a certain angle. Twin boundaries also can divide the matrix and generate grain refinement. At the same time, twin boundaries as two-dimensional lattice defects can be recrystallization nucleation sites, which facilitate recrystallization. It is evident that twinning can be used to improve the mechanical properties of Mg alloys. Zhang et al. [1] found that {10–12} twinning is one of the main deformation mechanisms of cast ZK60 Mg alloy during uniaxial compression at room temperature. It plays an important role in the evolution of microstructure, texture, and mechanical properties. Lu et al. [2] further developed a high-strength and -toughness Mg-Gd-Y alloy via MDIF (multidirectional impact forging) using {10–12} twin and correlated recrystallization. The forged sample had a fine-grained microstructure with an average grain size of ~5.7 µm and a weak nonbasal texture, and showed a high TYS (tensile yield strength) of 337 MPa, an EL (elongation) of 11.5%, and a ST (static toughness) of 50.4 MJ/m^3^. It also presented yield isotropy (the ratio of compression yield strength/tensile yield strength along the forging direction was ≈1.0). Wang et al. [3] investigated the flow behavior of solution-treated Mg-3.2Bi-0.8Ca (BX31, wt.%) alloy during hot compression under different deformation conditions, and made hot processing maps for confirming a suitable hot working range. With the assistance of a hot processing map, the as-extruded alloy exhibited a smooth surface, a fine DRX structure with weak off-basal texture, and good strength–ductility synergy. In addition, Zhang et al. [4] prepared Mg-3Sn-1Mn-xLa alloy bars using backward extrusion, and systematically studied the effects of the La content on the microstructure and mechanical properties of the alloy. With the addition of La, the Mg_2_Sn phases exhibited significant refinement and spheroidization, and the grain size was significantly refined. Therefore, the mechanical properties of the extruded Mg-3Sn-1Mn-xLa alloy were significantly enhanced. A Mg alloy rolling sheet usually exhibits a strong basal texture and bad formability. Wang et al. [5] applied multipass high-temperature cross-rolling with interpass annealing to Mg-3Y alloy. The Mg-3Y alloy sheet presented a complete SRXed microstructure consisting of uniform equiaxed grains and a weakened multiple-peak texture. Systemic characterization and analysis indicated that the enhanced activity of basal <a> slip and randomized grain orientation played a significant role in decreasing the anisotropy of the Mg-3Y alloy sheet, which contributed to the formation of high stretch formability (~6.2 mm) at room temperature.

Mg alloys have attracted great attention as promising biodegradable materials for orthopedic implants and cardiovascular interventional devices, but the degradation rate is unbalanced due to their poor corrosion resistance in a physiological environment, which seriously affects their clinical use. Adding alloying elements and surface modification technology are two main ways to improve the corrosion resistance of Mg alloys. From the perspective of corrosion resistance and the biocompatibility of biomedical magnesium alloy materials, Guo et al. [6] reviewed the application and characteristics of six different surface-coating modifications in the biomedical magnesium alloy field, including the chemical conversion method, microarc oxidation method, sol–gel method, electrophoretic deposition, hydrothermal method, and thermal spraying method, and looked ahead towards the development prospects of surface-coating modification. Moreover, Fu et al. [7] investigated the effect of Ca addition on the corrosion behavior of biodegradable Mg-4.0Zn-0.2Mn alloys in Hank’s solution. It was suggested that the Ca_2_Mg_6_Zn_3_ acted as a cathode to accelerate the corrosion process due to the microgalvanic effect.

Although it is generally believed that Mg alloys have excellent casting properties, the complexity of aerospace structures and imperfect casting technology require the usage of additive welding technologies. Shalomeev et al. [8] developed a scandium-containing filler metal from a Mg-Zr-Nd system alloy for the welding of aircraft castings. The proposed filler material composition with an improved set of properties for the welding of body castings from a Mg-Zr-Nd system alloy for aircraft engines makes it possible to increase their reliability and durability in general, extend the service life of aircraft engines, and gain economic benefits. As a combustible metal powder, Mg may also be used for the development of advanced weapons and equipment. Ma et al. [9] fabricated MgB_2_ via a combination of mechanical alloying and heat treatment, and found that its ignition temperature was greatly reduced in comparison with boron, which suggests that MgB_2_ may be used in gunpowder, propellant, explosives, and pyrotechnics due to its improved ignition performance. Additive manufacturing (AM) is largely capable of manufacturing structures with high complexities. Liu et al. [10] manufactured Al-Mg alloy walls via WAAM (wire and arc additive manufacturing), which showed better performance than those produced using the traditional casting process under the optimal process parameters.
